# Characteristics and Health Care Utilization of Different Segments of a Multiethnic Asian Population in Singapore

**DOI:** 10.1001/jamanetworkopen.2019.10878

**Published:** 2019-09-06

**Authors:** Shi Yan, Yu Heng Kwan, Julian Thumboo, Lian Leng Low

**Affiliations:** 1 Family Medicine Academic Clinical Program, Duke-NUS Medical School, Singapore; 2 Program in Health Services and Systems Research, Duke-NUS Medical School, Singapore; 3 Department of Rheumatology and Immunology, Singapore General Hospital, Singapore, Bukit, Singapore; 4 Department of Family Medicine and Continuing Care, Singapore General Hospital, Bukit, Singapore

## Abstract

**Importance:**

Descriptive population-level health data are critical components of the evidence base for population health policy. Human populations often display marked heterogeneity in their health status among subgroups of the population. The recent widespread adoption of electronic health records provides opportunities to use routine real-world health care data to examine population health.

**Objective:**

To report population sociodemographic characteristics, health conditions, health care utilization, and health care costs for different population segments of a multiethnic Asian population divided according to a modified British Columbia Population Segmentation Framework.

**Design, Setting, and Participants:**

This population-based cross-sectional study used 2016 data from the Singapore Eastern Regional Health System, the largest Regional Health System in Singapore. Data were obtained from deidentified national-level electronic health records at the Ministry of Health Singapore. Participants included all residents in the Singapore Eastern Regional Health System catchment area in 2016. The descriptive analysis was conducted in August 2018.

**Main Outcomes and Measures:**

Socioeconomic profiles, disease prevalence, health care utilization, and cost patterns of population segments.

**Results:**

The total size of the study population in 2016 was 1 181 024 residents (576 663 [48.83%] male; median [interquartile range] age, 40 [22-57] years). The population was divided into 8 segments: healthy with no outpatient utilization (493 483 residents), healthy with outpatient utilization (259 909 residents), healthy with inpatient admissions (49 588 residents), low complex (215 134 residents), medium complex (79 350 residents), high complex (44 445 residents), cancer (34 217 residents), and end of life (4898 residents). Overall, the 3 most prevalent conditions were chronic kidney disease (31.89%), hypertension (18.52%), and lipid disorders (18.33%). Distributions of chronic conditions differed across the segments. Different segments had varying health care utilization patterns: the high-complex segment had the highest number of primary care clinic visits (mean [SD], 4.25 [5.46] visits), the cancer segment had the highest number of specialist outpatient clinic visits (mean [SD], 5.55 [8.49] visits), and the end-of-life segment had the highest numbers of accident and emergency department visits (mean [SD], 1.80 [1.88] visits) and inpatient admissions (mean [SD], 1.99 [1.89] admissions) during 2016. For health care costs, specialist outpatient clinic and inpatient care together made up more than 85% of the total cost of nearly 2 billion Singapore dollars. The end-of-life segment contributed approximately 50% of the health care cost per capita of 60 000 Singapore dollars.

**Conclusions and Relevance:**

Different population segments displayed heterogeneity in sociodemographic characteristics, health conditions, health care utilization, and health care cost patterns. This critical health information can be used as baseline data to inform regional and national health priorities for health services research and policy.

## Key Points

**Question:**

What are the sociodemographic characteristics, health conditions, health care utilization, and health care costs for different population segments of a multiethnic Asian population in Singapore divided according to a modified British Columbia Population Segmentation Framework?

**Findings:**

In this cross-sectional study, the study population of 1 181 024 individuals was divided into 8 segments with distinct and heterogeneous sociodemographic characteristics, health conditions, health care utilization, and health care cost patterns.

**Meaning:**

This critical population-level health information can be used as baseline data to inform regional and national health priorities for health services research and policy.

## Teaser

This cross-sectional study using data from Singapore national health records examines the sociodemographic characteristics, health conditions, health care utilization, and health care cost patterns for different segments of a multiethnic Asian population.

## Introduction

Population health is an emerging concept that is defined as “the health outcomes of a group of individuals, including the distribution of such outcomes within the group.”^1^ Valid and reliable studies^2,3,4^ of the health states of populations are critical components of the evidence base for population health policy formulation, evaluation of health systems, and development of intervention programs. Interest in population health has been increasing in the medical literature, including many recent population-level studies.^5,6,7,8^ Population-based approaches to characterizing the complexity of people’s health, including the distribution, variations, and trends of diseases, will make a significant contribution to the field of population health.

Human populations often display marked heterogeneity in their health and disease status, not only between populations but also among subgroups of the same population.^9,10^ For example, within King County, Washington, there is an approximately 18-year difference in male life expectancy among the US Census tracts, even though the overall health in King County is among the best in the country, ranking in the 95th percentile among county health nationwide.^11^ Population health science has long focused on aggregate health indicators; however, the study of variability and heterogeneity can yield a deeper understanding of population health characteristics and needs.^12^ Although such research is challenging, it is important to identify the different health care needs and health characteristics of subgroups within a particular population.^13^

One promising approach to address the issue of population health heterogeneity is population segmentation. By dividing a heterogeneous population into somewhat homogeneous subgroups, population segmentation helps in understanding the distinctive needs of different parts of the population and developing care interventions and services that different subgroups of people might need.^14^ Since its conception by Lynn et al,^15^ population segmentation has been widely applied in many health systems, including British Columbia, Canada,^13^ Northwest London, United Kingdom,^16^ and Lombardy, Italy.^17^ However, data on population segmentation in Asian settings are sparse.

Traditionally, population health studies rely heavily on population-level surveys, such as the National Health Interview Survey by the US Census Bureau^18^ and National Population Health Survey by Statistics Canada.^19^ However, population-level surveys are labor intense and time-consuming and rely heavily on self-reported health measures. The interpretation and comparability of these measures are problematic when different persons understand and respond to a given question in different ways.^20^ The recent widespread adoption of electronic health records (EHRs) globally offers opportunities to use clinical data to examine population health because they provide real-world data available for analysis at any time. The use of EHRs not only facilitates clinical decision-making but also reduces the potential for inaccuracy and incompleteness of data entry and storage.^21^ In this population-level health study, we report population sociodemographic characteristics, health conditions, health care utilization, and health care costs of different population segments of a multiethnic Asian population divided according to a modified British Columbia Population Segmentation Framework.^13^

## Methods

### Data Source

This population-based cross-sectional descriptive study used routinely collected retrospective data from the Ministry of Health Singapore Division of Policy Research and Evaluation. We extracted the following data for each resident in the study population and presented population-level data in this study: demographic characteristics (age, sex, and race/ethnicity), disease diagnosis (as defined by diagnosis codes from the *International Classification of Diseases*,* Tenth Revision*), and health care utilization nationwide, including polyclinic visits, general practitioner visits, specialist outpatient clinic visits, accident and emergency department visits, and inpatient admissions. Polyclinics are large public primary care clinics located throughout the country that provide subsidized primary care, including medical treatment, preventive health care, and health education.^22^ Charlson Comorbidity Index^23^ and Elixhauser Comorbidity Index^24^ scores were also calculated for overall population and each population segment. The data sets captured comprehensive data and covered health facility visits to all health care professionals and to public health care institutions, all hospitalizations and outpatient operations in private hospitals, and outpatient visits that are covered under MediSave (Singapore’s national medical savings account program) and MediShield (Singapore’s national health insurance program). Data from general practitioners were included if they participate in Community Health Assist Scheme, a government program that enables Singapore citizens from low- and middle-income households and those born on or before December 31, 1949, to receive subsidies for medical and dental care.

This study was exempted from formal review by the SingHealth Centralized Institutional Review Board. The need for informed consent was waived because the data were deidentified. This study followed the Strengthening the Reporting of Observational Studies in Epidemiology (STROBE) reporting guideline for cross-sectional studies.^25^

### Study Population

The study population comprised all residents who lived in the Singapore Eastern Regional Health System (RHS) in 2016. The Eastern RHS is the largest RHS established by the Ministry of Health Singapore to integrate health service across health care systems in the Eastern region across care settings—primary care, intermediate care, long-term care, community-based care, and tertiary care—to deliver comprehensive care to the regional population in eastern Singapore.^26^ The catchment population as of June 2017 was approximately 1 336 840.^27^

### Population Segmentation

The population segmentation approach used in this study was based on the British Columbia Population Segmentation Framework.^13^ The framework was chosen and modified by a panel of local experts in Singapore who are senior clinicians and health policy leaders in the Eastern RHS and Ministry of Health Singapore. They come from a variety of specialties with extensive experience in health policy and clinical care. This ensures that policy implementation is relevant to the specific health care system setting within Singapore. The definitions and criteria for the modified British Columbia Population Segmentation Framework are finalized via focused group discussions among the Ministry of Health Singapore and Eastern RHS experts (Table 1).

**Table 1.** Modified British Columbia Population Segmentation Framework

Category	Description
End of life	Patients who died in the last 1 y and had received a diagnosis of high complex diseases and/or cancer
Cancer	Cancer or metastatic cancer
High complex	Dementia, heart failure, kidney transplant, severe liver disease OR had this event or intervention (stroke or dialysis) OR had this combination of conditions (angina and chronic obstructive pulmonary disease; acute myocardial infarction and predialysis chronic kidney disease; rheumatoid arthritis and osteoporosis; diabetes, hypertension, and osteoarthritis)
Medium complex	Predialysis chronic kidney disease, chronic obstructive pulmonary disease, coronary heart disease including angina, rheumatoid arthritis, Parkinson disease, atrial fibrillation, moderate liver disease, peripheral vascular disease, OR had this event or intervention (coronary artery bypass grafting, acute myocardial infarction, percutaneous coronary intervention, and lower extremity amputation) OR had this combination of conditions (osteoporosis and osteoarthritis, osteoporosis and hypertension, osteoarthritis and hypertension, osteoporosis and spine or hip fracture)
Low complex	Diabetes, hypertension, osteoporosis, osteoarthritis, asthma, epilepsy, hyperthyroidism, hypothyroidism, benign prostatic hypertrophy, and lipid disorders
Healthy with inpatient admissions	Not end of life, cancer, high, medium, or low complex but with ≥1 inpatient admission
Healthy with outpatient utilization	Not end of life, cancer, high, medium, or low complex but with ≥1 polyclinic or specialist outpatient clinic visit and no inpatient admissions
Healthy with no outpatient utilization	Not end of life, cancer, high, medium, or low complex with no polyclinic visit, specialist outpatient clinic visit, and inpatient admissions

### Statistical Analysis

The descriptive analysis was conducted in August 2018. Means, SDs, and percentages were used as appropriate to describe the data. Stata statistical software version 14.0 (StataCorp) was used for statistical analysis, and Tableau statistical software version 2018.2.2 (Tableau Software) was used for data visualization.

## Results

The total size of the study population in 2016 was 1 181 024 residents. As shown in Table 2, the population was divided into 8 segments—healthy with no outpatient utilization (493 483 residents), healthy with outpatient utilization (259 909 residents), healthy with inpatient admissions (49 588 residents), low complex (215 134 residents), medium complex (79 350 residents), high complex (44 445 residents), cancer (34 217 residents), and end of life (4898 residents)—on the basis of the modified British Columbia Population Segmentation Framework.^13^ The largest segment was healthy with no outpatient utilization (493 483 residents) and the smallest was end of life (4898 residents).

**Table 2.** Population Health Characteristics in 2016, by Population Segment

Characteristic	Total	Healthy With No Outpatient Utilization	Healthy With Outpatient Utilization	Healthy With Inpatient Admissions	Low Complex	Medium Complex	High Complex	Cancer	End of Life
Population size, No.	1 181 024	493 483	259 909	49 588	215 134	79 350	44 445	34 217	4898
Sociodemographic characteristics									
Male, No. (%)	576 663 (48.83)	249 375 (50.53)	123 180 (47.39)	19 964 (40.26)	102 232 (47.52)	44 908 (56.59)	21 746 (48.93)	12 743 (37.24)	2515 (51.35)
Age, median (IQR), y	40 (22-57)	33 (18-46)	28 (15-45)	29 (6-38)	55 (41-64)	63 (54-72)	69 (60-78)	63 (54-73)	78 (67-86)
Residential status, No. (%)									
Permanent resident	106 034 (8.98)	64 952 (13.16)	18 982 (7.30)	6447 (13.00)	9440 (4.39)	3268 (4.12)	1073 (2.41)	1746 (5.10)	126 (2.57)
Singapore citizen	1 060 860 (89.83)	420 324 (85.17)	236 848 (91.13)	42 131 (84.96)	205 272 (95.42)	75 952 (95.72)	43 228 (97.26)	32 395 (94.68)	4710 (96.16)
Ethnic group, No. (%)									
Chinese	827 811 (70.09)	338 184 (68.53)	179 704 (69.14)	33 520 (67.60)	154 052 (71.61)	58 463 (73.68)	31 881 (71.73)	28 318 (82.76)	3689 (75.32)
Indian	84 104 (7.12)	33 539 (6.80)	18 560 (7.14)	3679 (7.42)	16 154 (7.51)	6361 (8.02)	3880 (8.73)	1648 (4.82)	283 (5.78)
Malay	158 907 (13.46)	56 866 (11.52)	39 914 (15.36)	8062 (16.26)	32 636 (15.17)	10 813 (13.63)	7161 (16.11)	2903 (8.48)	552 (11.27)
Others	109 771 (9.29)	64 893 (13.15)	21 731 (8.36)	4273 (8.62)	12 214 (5.68)	3614 (4.55)	1391 (3.13)	1281 (3.74)	374 (7.64)
Community Health Assist Scheme status of not enrolled, No. (%)^a^	777 007 (65.79)	364 384 (73.84)	176 021 (67.72)	39 196 (79.04)	115 830 (53.84)	41 091 (51.78)	19 060 (42.88)	19 104 (55.83)	2321 (47.39)
Prevalence of diseases or conditions, No. (%)									
Angina	22 554 (1.91)	0	0	0	0	13 802 (17.39)	6354 (14.30)	1763 (5.15)	635 (12.96)
Anxiety	15 687 (1.33)	2317 (0.47)	3320 (1.28)	700 (1.41)	4822 (2.24)	2233 (2.81)	1309 (2.95)	845 (2.47)	141 (2.88)
Arrhythmia	18 371 (1.56)	1588 (0.32)	1485 (0.57)	410 (0.83)	2861 (1.33)	4804 (6.05)	4628 (10.41)	1519 (4.44)	1 076 (21.97)
Asthma	60 634 (5.13)	0	0	0	46 790 (21.75)	7072 (8.91)	4276 (9.62)	2078 (6.07)	418 (8.53)
Atrial fibrillation	14 316 (1.21)	0	0	0	0	6027 (7.60)	5675 (12.77)	1371 (4.01)	1243 (25.38)
Benign prostatic hyperplasia	22 322 (1.89)	0	0	0	8326 (3.87)	5803 (7.31)	4180 (9.40)	3236 (9.46)	777 (15.86)
Chronic obstructive pulmonary disease	10 447 (0.88)	0	0	0	0	6328 (7.97)	2560 (5.76)	983 (2.87)	576 (11.76)
Chronic heart disease	58 589 (4.96)	0	0	0	0	34 089 (42.96)	17 231 (38.77)	4832 (14.12)	2437 (49.76)
Diabetes	102 738 (8.70)	0	0	0	46 543 (21.63)	18 735 (23.61)	26 893 (60.51)	8222 (24.03)	2345 (47.88)
Lipid disorders	216 510 (18.33)	0	0	0	109 412 (50.86)	51 395 (64.77)	36 187 (81.42)	16 253 (47.50)	3263 (66.62)
Epilepsy	8429 (0.71)	0	0	0	5669 (2.64)	692 (0.87)	1354 (3.05)	459 (1.34)	255 (5.21)
Gestational diabetes	7633 (0.65)	1980 (0.40)	1500 (0.58)	957 (1.93)	2549 (1.18)	313 (0.39)	172 (0.39)	157 (0.46)	5 (0.10)
Hemorrhagic stroke	4978 (0.42)	0	0	0	0	0	4166 (9.37)	376 (1.10)	436 (8.90)
Heart failure	13 889 (1.18)	0	0	0	0	0	11 033 (24.82)	1281 (3.74)	1575 (32.16)
Hip fracture	6870 (0.58)	374 (0.08)	325 (0.13)	119 (0.24)	1158 (0.54)	1455 (1.83)	2174 (4.89)	680 (1.99)	585 (11.94)
Hypertension	218 767 (18.52)	0	0	0	102 831 (47.80)	55 927 (70.48)	39 044 (87.85)	17 079 (49.91)	3886 (79.34)
Hyperthyroidism	12 239 (1.04)	0	0	0	7990 (3.71)	1800 (2.27)	1396 (3.14)	883 (2.58)	170 (3.47)
Hypothyroidism	12 329 (1.04)	0	0	0	6460 (3.00)	2120 (2.67)	1859 (4.18)	1576 (4.61)	314 (6.41)
Insomnia	11 800 (1.00)	1405 (0.28)	2731 (1.05)	417 (0.84)	3599 (1.67)	1707 (2.15)	1136 (2.56)	655 (1.91)	150 (3.06)
Ischemic stroke	14 527 (1.23)	0	0	0	0	0	12 190 (27.43)	1254 (3.66)	1083 (22.11)
Myocardial infarction	19 312 (1.64)	0	0	0	0	8900 (11.22)	7518 (16.92)	1353 (3.95)	1541 (31.46)
Nephritis	5925 (0.50)	7 (0.00)^b^	41 (0.02)	21 (0.04)	311 (0.14)	3054 (3.85)	1784 (4.01)	526 (1.54)	181 (3.70)
Nephrosis	3145 (0.27)	9 (0.00)^b^	66 (0.03)	26 (0.05)	310 (0.14)	965 (1.22)	1286 (2.89)	301 (0.88)	182 (3.72)
Osteoarthritis	66 968 (5.67)	0	0	0	19 909 (9.25)	22 092 (27.84)	18 799 (42.30)	5128 (14.99)	1040 (21.23)
Osteoporosis	1811 (0.15)	0	0	0	127 (0.06)	641 (0.81)	660 (1.48)	222 (0.65)	161 (3.29)
Parkinson disease	3128 (0.26)	0	0	0	0	1599 (2.02)	983 (2.21)	286 (0.84)	260 (5.31)
Peripheral vascular disease	5196 (0.44)	0	0	0	0	2163 (2.73)	2062 (4.64)	439 (1.28)	532 (10.86)
Prediabetes	10 862 (0.92)	298 (0.06)	229 (0.09)	58 (0.12)	4031 (1.87)	2571 (3.24)	2479 (5.58)	937 (2.74)	259 (5.29)
Hypertension during pregnancy	1958 (0.17)	476 (0.10)	299 (0.12)	337 (0.68)	714 (0.33)	78 (0.10)	34 (0.08)	19 (0.06)	1 (0.02)
Psoriasis	2602 (0.22)	261 (0.05)	490 (0.19)	77 (0.16)	752 (0.35)	485 (0.61)	354 (0.80)	139 (0.41)	44 (0.90)
Renal disease	36 539 (3.09)	0	0	0	0	19 194 (24.19)	11 885 (26.74)	3437 (10.04)	2023 (41.30)
Respiratory failure	4870 (0.41)	104 (0.02)	93 (0.04)	56 (0.11)	442 (0.21)	859 (1.08)	1946 (4.38)	524 (1.53)	846 (17.27)
Rheumatoid arthritis	2895 (0.25)	0	0	0	0	2041 (2.57)	541 (1.22)	250 (0.73)	63 (1.29)
Secondary hypertension	410 (0.03)	8 (0.00)^b^	16 (0.01)	13 (0.03)	56 (0.03)	87 (0.11)	167 (0.38)	45 (0.13)	18 (0.37)
Spine fracture	4900 (0.41)	749 (0.15)	499 (0.19)	158 (0.32)	1056 (0.49)	846 (1.07)	990 (2.23)	404 (1.18)	198 (4.04)
Stroke	18 120 (1.53)	0	0	0	0	0	15 254 (34.32)	1527 (4.46)	1339 (27.34)
Weight loss	14 718 (1.25)	1023 (0.21)	1078 (0.41)	354 (0.71)	5787 (2.69)	2828 (3.56)	2733 (6.15)	765 (2.24)	150 (3.06)
Bipolar disorder	1762 (0.15)	215 (0.04)	357 (0.14)	131 (0.26)	555 (0.26)	200 (0.25)	195 (0.44)	90 (0.26)	19 (0.39)
Dementia	6977 (0.59)	0	0	0	0	0	5209 (11.72)	709 (2.07)	1059 (21.62)
General anxiety disease	1469 (0.12)	88 (0.02)	376 (0.14)	63 (0.13)	470 (0.22)	237 (0.30)	156 (0.35)	69 (0.20)	10 (0.20)
Major depression	21 655 (1.83)	3301 (0.67)	4139 (1.59)	1149 (2.32)	5789 (2.69)	2674 (3.37)	2901 (6.53)	1194 (3.49)	508 (10.37)
Schizophrenia	4942 (0.42)	516 (0.10)	1011 (0.39)	218 (0.44)	1736 (0.81)	640 (0.81)	535 (1.20)	223 (0.65)	63 (1.29)
Cancer without metastasis	36 022 (3.05)	0	0	0	0	0	0	33 647 (98.33)	2375 (48.49)
Metastatic carcinoma	10 382 (0.88)	0	0	0	0	0	0	8822 (25.78)	1560 (31.85)
Moderate liver disease	2514 (0.21)	0	0	0	0	770 (0.97)	889 (2.00)	541 (1.58)	314 (6.41)
Severe liver disease	1422 (0.12)	0	0	0	0	0	905 (2.04)	288 (0.84)	229 (4.68)
Coronary artery bypass graft	4760 (0.40)	0	0	0	0	2509 (3.16)	1759 (3.96)	338 (0.99)	154 (3.14)
Percutaneous coronary intervention	17 206 (1.46)	0	0	0	0	10 840 (13.66)	4789 (10.78)	1139 (3.33)	438 (8.94)
Chronic kidney disease, receiving dialysis or predialysis	3467 (0.29)	0	0	0	0	0	2534 (5.70)	432 (1.26)	501 (10.23)
Kidney transplant	99 (0.01)	0	0	0	0	0	95 (0.21)	3 (0.01)	1 (0.02)
Major lower extremity amputation	714 (0.06)	0	0	0	0	268 (0.34)	269 (0.61)	51 (0.15)	126 (2.57)
Minor lower extremity amputation	1341 (0.11)	0	0	0	0	575 (0.72)	549 (1.24)	76 (0.22)	141 (2.88)
Chronic kidney disease	376 590 (31.89)	48 451 (9.82)	58 063 (22.34)	18 267 (36.84)	121 512 (56.48)	61 020 (76.90)	39 456 (88.77)	25 331 (74.03)	4490 (91.67)
Charlson Comorbidity Index score, mean (SD)	0.65 (1.37)	0.10 (0.30)	0.22 (0.01)	0.37 (0.02)	0.78 (0.15)	1.76 (1.79)	3.08 (4.18)	4.95 (8.27)	6.75 (12.13)
Elixhauser Comorbidity Index score, mean (SD)	0.60 (2.56)	0.01 (0.46)	0.42 (0.65)	0.49 (0.82)	0.71 (1.22)	1.19 (2.91)	1.65 (5.24)	3.07 (6.19)	3.46 (8.13)
Health care facility visits, mean (SD), No.									
Polyclinic visits	1.51 (3.27)	0	1.87 (2.81)	1.60 (4.03)	2.91 (3.97)	3.73 (4.94)	4.25 (5.46)	2.88 (4.36)	1.27 (3.58)
Specialist outpatient clinic visits	1.11 (2.84)	0	1.23 (1.90)	2.21 (3.50)	1.42 (2.59)	2.54 (3.58)	3.79 (4.53)	5.55 (8.49)	4.10 (7.24)
Accident and emergency department visits	0.23 (0.82)	0.06 (0.30)	0.25 (0.75)	0.80 (1.38)	0.22 (0.78)	0.37 (1.04)	0.74 (1.98)	0.38 (1.07)	1.80 (1.88)
Inpatient admissions	0.13 (0.55)	0	0	1.23 (0.70)	0.11 (0.45)	0.26 (0.72)	0.61 (1.27)	0.46 (1.19)	1.99 (1.89)

Abbreviation: IQR, interquartile range.

^a^ Community Health Assist Scheme enables Singapore citizens from lower- to middle-income households, and all citizens born on or before December 31, 1949, to receive subsidies for medical and dental care.

^b^ Percentages were rounded to 0.00% because they were so small.

### Sociodemographic Characteristics

The median (interquartile range) age of the study population was 40 (22-57) years, and 576 663 participants (48.83%) were male. Table 2 shows that the segment with cancer had a disproportionately small percentage of male participants (37.24%). In the total population, most (70.09%) were ethnically Chinese. The proportion of Chinese participants was higher in the low-complex, medium-complex, high-complex, cancer, and end-of-life segments. These segments also had older participants and a higher proportion of residents enrolled in Community Health Assist Scheme compared with the overall population.

The Figure shows the proportion of patients in different age groups within each segment. Healthier segments had high proportions of patients younger than 44 years, whereas the complex, cancer, and end-of-life segments had high proportions of patients between ages 45 and 84 years. The end-of-life segment had the highest proportion of patients aged 65 years and older.

**Figure.**
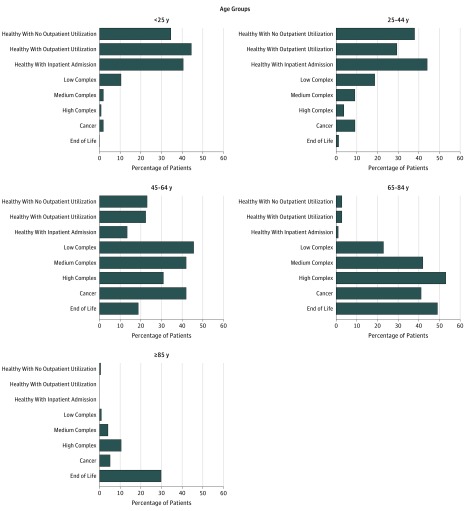
Proportion of Patients in Different Age Groups Within Each Segment Graphs show proportion of patients in different age groups within each segment. Healthier segments have high proportions of patients younger than 44 years, whereas the complex, cancer, and end-of-life segments have high proportions of patients ages 45 to 84 years. The end-of-life segment has the highest proportion of patients age 65 years and older.

### Health Conditions

Overall, the 5 most prevalent diseases were chronic kidney disease (31.89%), hypertension (18.52%), lipid disorders (18.33%), diabetes (8.70%), and osteoarthritis (5.67%). In Table 2, for the first 3 segments (healthy with no outpatient utilization, healthy with outpatient utilization, and healthy with inpatient admissions), the prevalence of chronic diseases was generally lower than that in the overall population. The other 5 segments (low complex, medium complex, high complex, cancer, and end of life) had higher chronic disease prevalence compared with the population mean. For most of the diseases, prevalence increased as population segments became more complex, from the healthy with no outpatient utilization segment to the end-of-life segment. Similar trends were observed with Charlson Comorbidity Index and Elixhauser Comorbidity Index scores.

The distribution of chronic conditions across the different segments is shown in Table 3. As expected, most diseases concentrated in specific segments according to the modified British Columbia Population Segmentation Framework.^13^ For example, cancer without metastasis was defined under the cancer category in the modified British Columbia Population Segmentation Framework, as shown in Table 1, and 93.41% of patients with cancer without metastasis were classified into the cancer segment. Some diseases were distributed more widely into multiple segments. For example, although osteoarthritis was defined under the low-complex category, 32.99% of patients with osteoarthritis were in the medium-complex segment and another 28.07% were in the high-complex segment.

**Table 3.** Distribution of Chronic Conditions Listed in the Modified British Columbia Population Segmentation Framework, by Population Segment

Condition	Patients, %^a^				Patients, No.
	Medium Complex	High Complex	Cancer	End of Life	
Minimum criteria for low complex chronic conditions					
Diabetes	18.24	26.18	8.00	2.28	102 738
Hypertension	25.56	17.85	7.81	1.78	218 767
Osteoporosis	35.39	36.44	12.26	8.89	1811
Osteoarthritis	32.99	28.07	7.66	1.55	66 968
Asthma	11.66	7.05	3.43	0.69	60 634
Epilepsy	8.21	16.06	5.45	3.03	8429
Hyperthyroidism	14.71	11.41	7.21	1.39	12 239
Hypothyroidism	17.20	15.08	12.78	2.55	12 329
Benign prostatic hyperplasia	26.00	18.73	14.50	3.48	22 322
Lipid disorders	23.74	16.71	7.51	1.51	216 510
Minimum criteria for medium complex chronic conditions					
Chronic kidney disease receiving dialysis or predialysis	0.00	73.09	12.46	14.45	3467
Chronic obstructive pulmonary disease	60.57	24.50	9.41	5.51	10 447
Coronary heart disease	58.18	29.41	8.25	4.16	58 589
Angina	61.20	28.17	7.82	2.82	22 554
Rheumatoid arthritis	70.50	18.69	8.64	2.18	2895
Parkinson disease	51.12	31.43	9.14	8.31	3128
Atrial fibrillation	42.10	39.64	9.58	8.68	14 316
Moderate liver disease	30.63	35.36	21.52	12.49	2514
Peripheral vascular disease	41.63	39.68	8.45	10.24	5196
Coronary artery bypass graft	52.71	36.95	7.10	3.24	4760
Myocardial infarction	46.09	38.93	7.01	7.98	19 312
Percutaneous coronary intervention	63.00	27.83	6.62	2.55	17 206
Major lower extremity amputation	37.54	37.68	7.14	17.65	714
Minor lower extremity amputation	42.88	40.94	5.67	10.51	1341
Minimum criteria for high complex chronic conditions					
Dementia		74.66	10.16	15.18	6977
Heart failure		79.44	9.22	11.34	13 889
Kidney transplant		95.96	3.03	1.01	99
Severe liver disease		63.64	20.25	16.10	1422
Stroke		84.18	8.43	7.39	18 120
Minimum criteria for cancer					
Cancer without metastasis			93.41	6.59	36 022
Metastatic carcinoma			84.97	15.03	10 382
Total	20.99	11.76	9.05	1.30	378 044

^a^ Each percentage represents the proportion of patients across the segments who received a diagnosis of that chronic condition (ie, each row adds up to 100%). The healthy with no outpatient utilization, healthy with outpatient utilization, and healthy with inpatient admissions segments are not included in this table because the percentages for all of them were 0.

### Health Care Utilization

As shown in Table 2, overall, residents in the Eastern Singapore RHS had a mean (SD) of 1.51 (3.27) polyclinic visits, 1.11 (2.84) specialist outpatient clinic visits, 0.23 (0.82) emergency department visit, and 0.13 (0.55) inpatient admission in 2016. The high-complex segment had the highest number of polyclinic visits (mean [SD], 4.25 [5.46] visits). The cancer segment had the highest specialist outpatient clinic visits (mean [SD], 5.55 [8.49] visits). The end-of-life segment had a mean (SD) of 1.80 (1.88) emergency department visits and 1.99 (1.89) inpatient admissions, both of which were the highest among all segments.

### Health Care Cost

The distribution of health care cost in 2016 across different segments is shown in Table 4. The healthy with inpatient admission segment accounted for the largest proportion of total cost (21.56%), even though its population size was less than 5% of the total population. Specialist outpatient clinic and inpatient admission costs together accounted for more than 85% of the total cost of nearly 2 billion Singapore dollars.

**Table 4.** Distribution of Health Care Cost in 2016, by Population Segment

Segment	Patients, No. (%)	Total Polyclinic Visit Cost, SGD^a^	Total GP Visit Cost, SGD^a^	Total Specialist Outpatient Clinic Visit Cost, SGD^a^	Total Accident and Emergency Department Visit Cost, SGD^a^	Total Inpatient Admission Cost, SGD^a^	Total Utilization Cost, SGD^a^	Total Utilization Cost by Segment, %
Healthy with no outpatient utilization	493 483 (41.78)	0	4.82	0	8.55	0	13.37	67
Healthy with outpatient utilization	259 909 (22.01)	31.27	3.77	109.13	19.49	0	163.65	8.18
Healthy with inpatient admissions	49 588 (4.20)	5.89	0.57	36.17	11.73	376.89	431.24	21.56
Low complex	215 134 (18.22)	50.73	19.64	101.39	16.52	149.95	338.22	16.91
Medium complex	79 350 (6.72)	28.60	10.17	65.83	11.09	182.53	298.22	14.91
High complex	44 445 (3.76)	21.97	6.69	55.20	12.92	247.58	344.35	17.22
Cancer	34 217 (2.90)	8.97	3.43	75.55	4.75	176.06	268.76	13.44
End of life	4898 (0.41)	0.54	0.35	8.73	3.53	128.90	142.05	7.10
Total	1 181 024 (100)	147.98	49.44	451.99	88.57	1261.90	1999.87	100.00
Total utilization cost by service, %		7.40	2.47	22.60	4.43	63.10	100.00	

Abbreviations: GP, general practitioner; SGD, Singapore dollars.

^a^ All costs are shown in millions of SGD (1 US dollar = 1.4465 SGD, as of December 31, 2016), incurred before government subsidies.

The distribution of health care cost per capita in 2016 across different segments is illustrated in eTable 1 in the Supplement. Health care costs per patient in the end-of-life segment were the largest (approximately 50%, or 60 000 Singapore dollars) compared with the other segments.

The distribution of health care cost per capita in 2016 in different age groups across segments is shown in eTable 2 in the Supplement. There seemed to be a general trend that older patients had higher health care cost per capita. Per capita cost for patients in the low-complex segment and healthy with or without outpatient utilization segments were generally low across all age groups. Patients in the end-of-life segment had high health cost per capita across all age groups.

## Discussion

In this article, we describe the heterogeneous health profiles of population segments in the Singapore Eastern RHS based on real-world data from routine EHRs. We found that different segments within a population have different sociodemographic characteristics, health conditions, health care utilization, and health care costs. This article provides a critical overview of and baseline information on health status at a population level to inform population health management. For example, compared with the Singapore National Health Survey, which reported a crude prevalence of diabetes of 11.3% among Singapore residents aged 18 to 69 years in 2010,^28^ our data show that the prevalence of diabetes among residents in the Singapore Eastern RHS was 8.70% in 2016. The lower diabetes prevalence might be multifactorial, including recent national-level concerted health promotion efforts by the Ministry of Health Singapore and different data-collection methods. The data presented here may serve as a starting point for further population health monitoring, care intervention, and policy evaluation. Future research opportunities include tracking the longitudinal health care utilization and cost of these population segments, examining the predictive ability of segment membership on health care utilization, and analyzing how individuals move between the population segments in the long term.

The present study adds to the emerging field of using real-world health data by demonstrating that routine clinical care data can be used for population health profiling and monitoring. Recently, there has been substantial enthusiasm for using routine health care data to provide real-world evidence; however, the widespread use of EHRs, administrative databases, and smart devices has heretofore not led to wide use of these data for clinical insights at the population health level, despite the obvious advantages of using routine care data, which is that the use of such data is both pragmatic and generalizable.^29^ Real-world data provide useful information that may yield new insights and stimulate new research questions and aid in the design of other interventional studies, such as a clinical trial, and facilitate policy making and practice.^29,30^ For example, our study found that the healthy with inpatient admission segment unexpectedly accounted for the largest proportion of total cost (21.56%), even though its population size was less than 5% of the total population. This finding warrants further research efforts in understanding the mechanism behind this phenomenon. One explanation for the participants’ health behavior is their private health insurance plan status, which might lead to higher inpatient service rates even though they do not have complex chronic conditions.

The optimal framework for and measures of population health status have long been debated. It is recognized that traditional health indicators alone, such as life expectancy, may not be sufficient for measuring and monitoring population health.^31^ Functional, psychosocial, quality-of-life, and behavioral variables have been proposed and used as important population health indicators.^32^ The US Institute of Medicine has also given serious attention to population health measurement and encouraged summary measures that include health-related quality-of-life data.^33^ Furthermore, a population health perspective also requires attention to resource utilization and allocation.^1^ Some summary measures have been developed to include a broad spectrum of health indicators in different policy and research contexts, such as EQ-5D^34^ and the Health Utilities Index.^35^ Although these data are often made available via population-based health surveys, they may not be routinely collected and stored in many health systems, including our data sets. To further complicate this matter, there is heterogeneity in data definition, collection, and reporting in the current medical literature of population health policy. One of the more widely accepted models is the Organisation for Economic Co-operation and Development health indicators framework,^36^ which reports health-related data in the following 5 categories: health status, risk factors for health, access to care, quality and outcome of care, and health care resources. However, controversy remains over what the universally important, relevant, and practical health indicators and performance measures are that researchers and policy makers should use, both conceptually and at the implementation level. This is likely dependent on each health care system’s policy priority within their economic and political context, objectives of the funding policy, and data availability.

### Limitations

This study has limitations. First, it is retrospective and relies on existing data sources. Second, our study may not be generalizable to other health care system settings because they may not be collecting the exact same type of data that we presented and, thus, may not find our study readily replicable; however, they may find our segmentation framework useful as a reference for their policy needs. Third, our database does not include patients who are exclusively managed in the private health care sector, which was small because MediSave is Singapore’s mandatory national medical savings account program and MediShield is Singapore’s national health insurance program that automatically covers all Singapore residents.

Population health measures need to be developed and validated in close consultation with stakeholders, taking local policy context and priorities into considerations. It remains a critical task for the field of population health research to determine what to measure and how to measure it, whether by population surveys or routine clinical databases. Future research efforts are ongoing to link our routine health care data with collaborators’ databases that are rich in social and behavioral data to further refine population segmentation frameworks.

## Conclusions

There are wide differences in the health characteristics of the Eastern RHS population in Singapore. Different population segments displayed heterogeneity in sociodemographic characteristics, health conditions, health care facility utilization, and cost. These data can be used to inform regional and national health priorities for health services research and policy.

## Notes

Supplement. **eTable 1.** Distribution of Health Cost Per Capita in 2016 Across Different Segments **eTable 2.** Distribution of Health Cost Per Capita in 2016 in Different Age Groups, Across Segments
