# Supplementary material for: Characteristics and Health Care Utilization of Different Segments of a Multiethnic Asian Population in Singapore
Source: JAMA Netw Open. 2019 Sep 6;2(9):e1910878. doi: 10.1001/jamanetworkopen.2019.10878 (PMC6735407; doi:10.1001/jamanetworkopen.2019.10878)
Supplement: Supplement. — eTable 1. Distribution of Health Cost Per Capita in 2016 Across Different Segments eTable 2. Distribution of Health Cost Per Capita in 2016 in Different Age Groups, Across Segments [file jamanetwopen-2-e1910878-s001.pdf]

Supplementary Online Content

Yan S, Kwan YH, Thumboo J, Low LL. Characteristics and health care utilization of different segments of a multiethnic Asian population in Singapore. *JAMA Netw Open*. 2019;2(9):e1910878. doi:10.1001/jamanetworkopen.2019.10878

eTable 1. Distribution of Health Cost Per Capita in 2016 Across Different Segments
eTable 2. Distribution of Health Cost Per Capita in 2016 in Different Age Groups, Across Segments

This supplementary material has been provided by the authors to give readers additional information about their work.

© 2019 Yan S et al. *JAMA Network Open.*

eTable 1. Distribution of Health Cost Per Capita in 2016 Across Different Segments

Segments (Costs per capita)	N	(%)	Total polyclinic visit cost	Total GP visit cost	Total specialist outpatient clinic visit cost	Total A&E visit cost	Total inpatient admission cost	Total utilization costs	(%)
Healthy with no outpatient utilisation	493,483	41.78%	$0.00	$9.77	$0.00	$17.32	$0.00	$27.10	0.05%
Healthy with outpatient utilisation	259,909	22.01%	$120.30	$14.49	$419.87	$74.97	$0.00	$629.63	1.06%
Healthy with inpatient admissions	49,588	4.20%	$118.88	$11.47	$729.32	$236.52	$7,600.35	$8,696.54	14.67%
Low complex	215,134	18.22%	$235.79	$91.27	$471.29	$76.81	$696.99	$1,572.15	2.65%
Medium complex	79,350	6.72%	$360.45	$128.20	$829.61	$139.71	$2,300.36	$3,758.34	6.34%
High complex	44,445	3.76%	$494.28	$150.46	$1,242.02	$290.72	$5,570.38	$7,747.87	13.07%
Cancer	34,217	2.90%	$262.23	$100.30	$2,207.87	$138.68	$5,145.40	$7,854.47	13.25%
End of Life	4,898	0.41%	$111.25	$71.31	$1,781.73	$720.69	$26,316.89	$29,001.88	48.92%
Total	1,181,024	100%	$1,703.19	$577.28	$7,681.72	$1,695.43	$47,630.37	$59,287.98	100%
Utilization share of estimated cost			2.87%	0.97%	12.96%	2.86%	80.34%	100.00%	
	**N(%)**								
	≤ 10%	11-20 %	21-30 %	31-40%	> 40%				

All costs are in SGD, incurred before subsidies. (1 USD= 1.4465 SGD, as of 31 December 2016).

© 2019 Yan S et al. *JAMA Network Open.*

eTable 2. Distribution of Health Cost Per Capita in 2016 in Different Age Groups, Across Segments

	Age groups										
	**Age < 25**		**Age 25 - 44**		**Age 45 - 64**		**Age 65 - 84**		**Age ≥ 85**		**All ages**
Healthy with no Outpatient Utilization	$42.05		$20.47		$15.61		$31.37		$7.02		$27.10
Healthy with Outpatient Utilization	$601.29		$622.20		$667.74		$827.85		$981.97		$629.63
Healthy with Inpatient Admissions	$6,140.27		$10,217.18		$10,926.06		$12,512.83		$12,409.31		$8,696.54
Low Complex	$1,213.21		$1,667.36		$1,466.01		$1,829.76		$2,346.17		$1,572.15
Medium Complex	$3,315.99		$2,833.45		$3,612.05		$4,028.37		$4,709.17		$3,758.34
High complex	$9,542.66		$8,137.35		$7,877.84		$7,614.75		$7,688.49		$7,747.87
Cancer	$12,656.13		$7,741.55		$7,244.22		$8,216.81		$8,259.42		$7,854.47
End of Life	$131,224.45		$43,130.77		$43,178.10		$29,399.01		$17,347.52		$29,001.88

**Cost per capita**				
≤ $2500	$2,501-5,000	$5,001-10,000	$10,001-20,000	>$20,000

All costs are in SGD, incurred before subsidies. (1 USD= 1.4465 SGD, as of 31 December 2016)

© 2019 Yan S et al. *JAMA Network Open.*
